# Influence of Diabase Filler on the Structure and Tribological Properties of Coatings Based on Ultrahigh Molecular Weight Polyethylene

**DOI:** 10.3390/polym15163465

**Published:** 2023-08-18

**Authors:** Mazhyn Skakov, Moldir Bayandinova, Igor Ocheredko, Baurzhan Tuyakbayev, Makpal Nurizinova, Alexander Gradoboev

**Affiliations:** 1 National Nuclear Center of the Republic of Kazakhstan, Ministry of Energy of the Republic of Kazakhstan, Kurchatov 071100, Kazakhstan; skakovmk@mail.ru; 2 National Scientific Laboratory of Collective Use, Sarsen Amanzholov East Kazakhstan University, Ust-Kamenogorsk 070000, Kazakhstan; egor007kz@mail.ru (I.O.); tu_baur1980@mail.ru (B.T.); makpal.nurizinova@gmail.com (M.N.); 3 Experimental Physics Department, Tomsk Polytechnic University, Tomsk 634050, Russia; gradoboev1@mail.ru

**Keywords:** thermal spraying, ultrahigh molecular weight polyethylene, diabase filler, wear resistance, microhardness

## Abstract

This article presents the results of a study of a composite coating made of ultrahigh molecular weight polyethylene (UHMWPE) with a diabase filler obtained by flame spraying. Diabase of 10 wt.%, 20 wt.%, 30 wt.% and 40 wt.% was chosen as a filler. The polymer coating was applied to the St3 metal substrate using temperature control in a conventional flame spraying process. The coating was studied using scanning electron microscopy, X-ray phase analysis, infrared spectroscopy, abrasive wear resistance, microhardness testing and determination of the friction coefficient. It has been shown that diabases do not have a negative effect on the initial chemical structure of UHMWPE and it is not subjected to destruction during flame spraying. The introduction of diabase into the composition of UHMWPE with a content of 10–40% of the total mass does not adversely affect the crystalline structure of the coating. It has been established that with an increase in the volume of the diabase filler, the wear resistance of the composite coating based on UHMWPE increases. It has been determined that with the addition of diabase, the microhardness of the coatings increases.

## 1. Introduction

As is known, polymer composite materials are widely used in modern technology, including engineering, transport and chemical industries, as well as in medicine. A special place is occupied by ultrahigh molecular weight polyethylene (UHMWPE) due to a peculiar combination of practically important properties—high wear resistance, hardness and low friction coefficient [1]. An additional perspective of its use in various branches of mechanical engineering is provided by the introduction of various fillers, which makes it possible to significantly improve the mechanical and tribological properties of composite materials based on it. One of the ways to solve this material science problem is the functionalization of fillers.

The introduction of some fillers makes it possible to form composite materials with enhanced mechanical and tribological properties, as well as to expand their areas of application [2].

Modern materials science is developing along the path of improving the multifunctionality of materials using surface electron beam processing [3,4] as well as by introducing special filler additives [5]. To improve the properties of coatings based on UHMWPE, various approaches have been developed, among which are the creation of composite materials [6], reinforcement with carbon fibers [7,8], nanoparticles [9] and carbon nanotubes [10], modification with oxygen plasma [11] and other composites [12]. In the last few years, researchers [13,14] have attempted to study the effect of fillers in the form of nanostructured Al_2_O_3_, ZrO_2_ powders and graphene plates on the tribological properties of UHMWPE-based composites. It has been established that an effective way to improve the physical and mechanical characteristics of coatings based on UHMWPE is the introduction of a modifier from ultrafine powders of aluminum and zirconium oxides, which have undergone preliminary processing using powder metallurgy methods and the optimal values of the volume fraction of modifiers that increase wear resistance by several times have been determined [13]. New composite coatings of ultrahigh molecular weight polyethylene with graphene filler were successfully fabricated using flame spraying [14]. The content of graphene was selected in the range of up to 1.0 wt.%. The UHMWPE + graphene composites did not undergo significant thermal degradation during coating. The addition of graphene resulted in an increase in microhardness and improved antiwear performance of UHMWPE coatings. This paper shows that graphene retained its nanolayer structure and contributed to a significant reduction in the coefficient of friction of the coatings.

When developing composite materials based on UHMWPE matrices, it is necessary to take into account the preferred conditions for their use:

(i) abrasion (e.g., lining of conveyors and trolleys);
(ii) dry sliding friction (geared wheels);

During operation in the field, products made from UHMWPE matrices may be subject to the above types of wear. In this regard, it is advisable to use fillers that can improve wear resistance against various types of wear as well as provide higher strength, lower coefficient of friction, etc. In this case, various dispersed materials are used to fill polymers [15].

At the same time, apparently, it is necessary to further search for new, commercially available materials—fillers, the introduction of which significantly improves the physical and mechanical properties of the composite based on UHMWPE. It is known [16] that the reinforcement of the UHMWPE matrix with basalt particles is effective in dry sliding friction: the wear resistance of composites increases by a factor of 3 with the addition of 20 wt.% filler. The addition of basalt fibers to UHMWPE provides an increase in abrasive wear resistance, which increases by a factor of 2.5 with a change in the mass fraction of the filler in the range of 10–20 wt.%. Samples of polymer composites were produced by hot isostatic pressing at a specific pressure of 10 MPa and a sintering temperature of 200 °C, followed by a cooling rate of 1.5 °C/min.

At the same time, diabase as a mineral raw material, close to basalt, has good mechanical properties and chemical resistance to aggressive environments, an affordable cost and environmental friendliness. Diabase is a volcanic igneous rock, the main component of which is well-preserved plagioclase, colorless and transparent and rare pyroxene. Its melting point is 1005–1250 °C [17].

However, in our opinion, the effect of a filler in the form of diabase on the structure and properties of coatings based on UHMWPE obtained using flame deposition has not yet been studied.

In this regard, the purpose of this work is to determine the effect of diabase as a filler on the structure and tribological characteristics of a coating based on UHMWPE obtained using flame deposition.

## 2. Materials and Methods

### 2.1. Materials

A commercial UHMWPE powder with a density of 930 kg/m^3^, a molecular weight of 2 10^6^ mol^−1^, T_melt_ = 135–150 °C and a bulk density of >0.4 g/cm^3^ was chosen as the main material for obtaining coatings.

And as a filler, a mineral diabase powder of the following content was chosen: 10 wt.%, 20 wt.%, 30 wt.% and 40 wt.%, the average particle size of diabase is 14 μm with a bulk density of ~1.1 g/cm^3^. In order to obtain a homogeneous composition of the UHMWPE composite with diabase filler, mechanical mixing of the initial powders was carried out in a ball mill for about 2 min; the rotation frequency was 1500 rpm.

### 2.2. Coating

Coatings of UHMWPE + diabase were deposited using the gas-flame method developed by us on a substrate made of St3 steel with dimensions of 70 mm × 70 mm × 2 mm [18]. The steel substrates were sandblasted before spraying. The atomizer used a multifunctional burner, propane was used as a fuel gas and air was used as an oxidizer [19]. When the feeder was fully loaded, the pressure gradually increased to reach the required technological height of 400 mm. In this case, the height of the fluidized bed gradually increased in proportion to the pressure. The fluidized bed reached the required height at a pressure of 0.13 MPa and the volume flow was 65 m^3^/h. The powder feed rate was 1 kg/min and the spray distance was 800 mm.

### 2.3. Research Methods

The microstructure of the initial powders and coatings made of UHMWPE with filler was studied using scanning electron microscopy on a CrossBeam XB 540 (Carl Zeiss, Germany) with an INCA Energy energy-dispersive microanalysis system. The working distance between the sample surface and the lower part of the objective was 4.7 mm and the accelerating voltage was 20 kV. Before the SEM study, the surfaces of the samples under study were covered with a conductive Au film.

X-ray phase analysis of the powders and the composite polymer was carried out on an Xpert PRO PANalytical instrument. During the study, a voltage of 40 kV and a current of 30 mA were applied to the anode copper tube, Cu-Kα radiation (λ = 1.541 Å) in the range of angles 2θ; the shooting step was 0.02 and the counting time was 0.5 s/step. Phase analysis of the obtained lines of diffraction patterns was carried out using the HighScore Plus and Mach 3 software packages (version powdercell 2.4). Sample preparation, selection of shooting modes and calculation of diffraction patterns were carried out according to the methods described in [20].

The degree of crystallinity (χ) was determined using the method described in [21] and the range of diffraction angles 2θ was 15–35°, which characterizes the ratio of the crystalline and amorphous phases in the polymer, which was calculated using the formula:

χ = S_cr_/S_a_, (1)

where S_cr_ is the area of the crystalline part (above the halo); S_a_ is the area of the amorphous part (under the halo).

The thermal characteristics of powders and coatings of UHMWPE + diabase were determined using differential scanning calorimetry (DSC) in the range of 10–40 wt.%. The measurements were carried out in accordance with the requirements of GOST R 55134-2012 and described in the instruction manual for the Labsys Evo instrument (Setaram, France) in the temperature range of 30–500 °C. The crystallization and melting behavior of the initial mixture of diabase-filled UHMWPE powders and the diabase-filled UHMWPE composite coating were studied in a nitrogen flow. Samples weighing ~10 mg were weighed and sealed in aluminum cups. Before being scanned by heating and cooling, the samples were melted at 500 °C and kept at this temperature for 5 min to remove the thermal history of the material. Then they were cooled from 500 to 30 °C at a rate of 10°C/min and subsequently heated from 30 to 500 °C at a rate of 10 °C/min.

The chemical composition and molecular structure of the composite coatings were studied using a Fourier transform infrared spectrometer (FTIR-801 Simex) with a resolution of 1 cm^−1^ in the range of 450–3500 cm^−1^ in accordance with the standard method and using auxiliary equipment for measuring the attenuated total reflection (ATR) and specular-diffuse reflection (SDR) at a temperature of 25+/−10C [22].

The thermal characteristics of UHMWPE powders and their mixture with diabase, UHMWPE + diabase coatings were studied on a LabSysevo differential thermogravimetric analyzer (Setaram, France) in an argon atmosphere. The temperature range was 30 ± 5–600 ± 5 °C at a heating rate of 10 ± 1 °C/min. The weight of the samples was approximately 20–40 mg.

The wear resistance of the polymer coatings was determined as the weight loss per unit of time in the process of abrasive wear. The tests were carried out on an abrasion test facility using the method described in [13]. The test conditions were: load 18 N, exposure time 10, 20, 30 min., abrasive material—corundum powder with grain size <100 microns.

The coefficient of friction was determined on a universal tribometer TRB³ (Anton Paar, Austria) according to the “ball-disk” friction scheme by sliding the UHMWPE coating samples with a filler without lubrication along the steel plane of the counter body with a linear velocity of 0.05 m/s at a room temperature of 25 ± 1 °C. The vertical load was 10 N. The counter body material was 100Cr_6_. The coefficient of friction of the tested materials was determined after passing the friction path (L) equal to 100 m [23]. Wear marks and roughness of the friction surface of the samples were examined using a Mitutoyo model Surftest 410 profilometer. The area of the friction track was determined using the Rhino Ceros 3.0 software by manually extracting the contour of the abrasion surface (friction track) and subsequent automatic calculation using image processing methods.

The microhardness of the samples was determined on a Metolab 502 instrument according to the Vickers method [24]. The measurement parameters were: load 0.025 g, exposure time 10 s. The Vickers number (HV) was calculated using the formula:

(2) $$\mathrm{HV}=1.854\frac{P}{d^{2}}$$

where *P* is the applied load and *d* is the average diagonal of the indent.

## 3. Results and Discussion

### 3.1. Results of SEM Analysis of Powders

**Figure 1:**
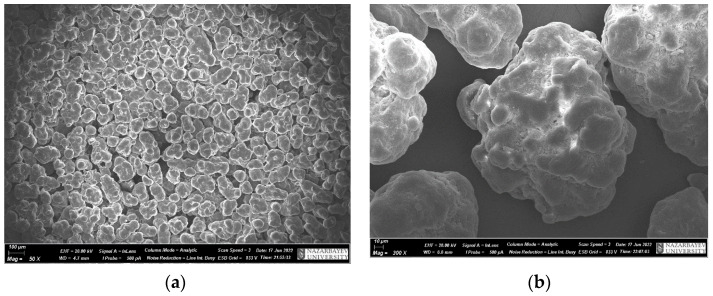

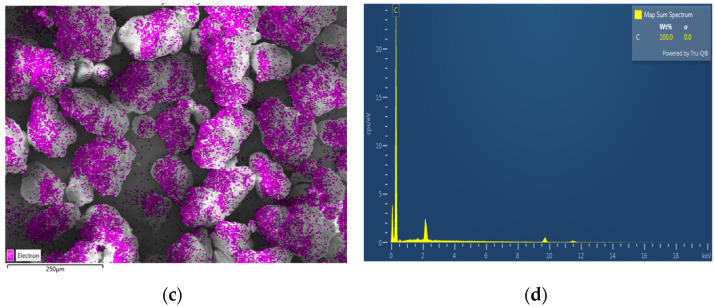
SEM images of UHMWPE powder (**a**,**b**), spectra for chemical analysis (**c**), elemental analysis of UHMWPE (**d**).

UHMWPE is a white powder, the morphology of which is shown in Figure 1, with an average particle size of 150 µm and it has a spherical shape. Energy-dispersive analysis shows the presence of only the carbon spectrum.

**Figure 2:**
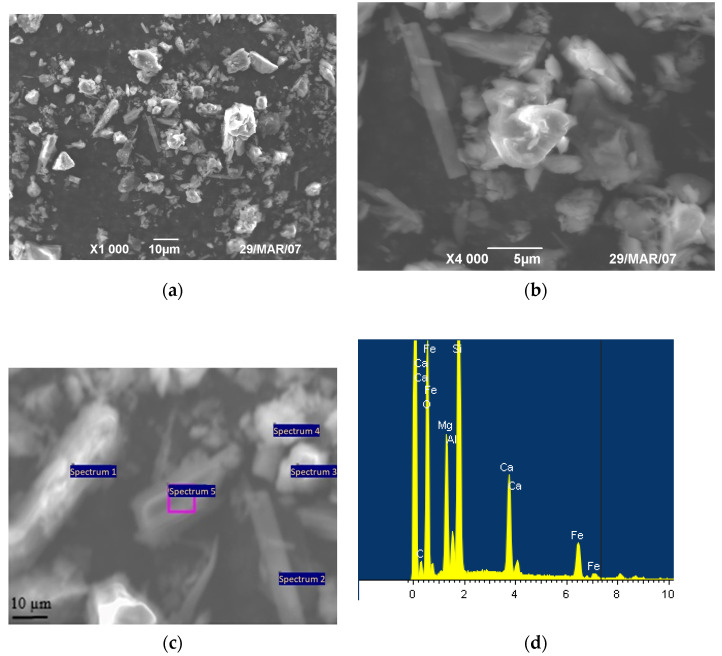
SEM images of the initial diabase powder (**a**,**b**), spectra for chemical analysis (**c**) elemental analysis of one spectrum of the diabase powder (**d**).

SEM showed that the structure of the diabase powder is of a mixed type (Figure 2); there are particles of lamellar, needle-like and spherical shapes.

The results of the analysis of the chemical composition and microstructure of the diabase powders and the data obtained are shown in Figure 2 and Table 1. The elemental composition of the spectrum 1 diabase powder is shown in Figure 2c. The chemical composition of the remaining spectra are shown in Table 1.

From the analysis of the results of the study presented in Table 1, it should be noted that the microstructure of the diabase powder contains a high content of reactive minerals in the form of silicon dioxide SiO_2_, iron oxide Fe_3_O_4_, calcium peroxide CaO, magnesium oxide MgO, aluminum oxide Al_2_O_3_, etc.

### 3.2. Results of SEM—Analysis of Coatings

**Figure 3:**
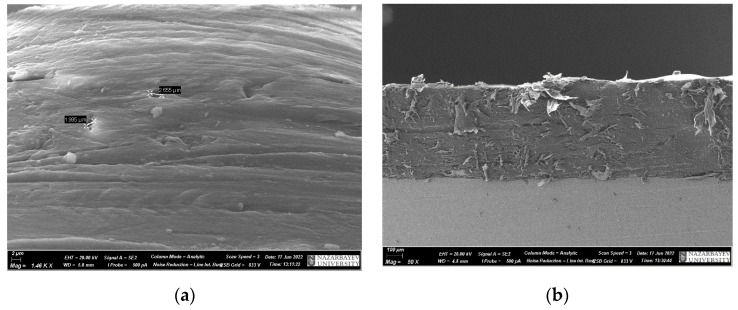
SEM images of UHMWPE coating morphology (**a**), coating cross-section (**b**).

Figure 3 shows the results of scanning electron microscopy of samples—UHMWPE coatings. In addition to the absence of cracks or other surface defects, no visible pores were observed in the coatings (Figure 3a), which presumably indicates the possibility of applying coatings based on UHMWPE using flame spraying.

As can be seen from Figure 1b, there are no delaminations of the polymer coating with the substrate.

**Figure 4:**
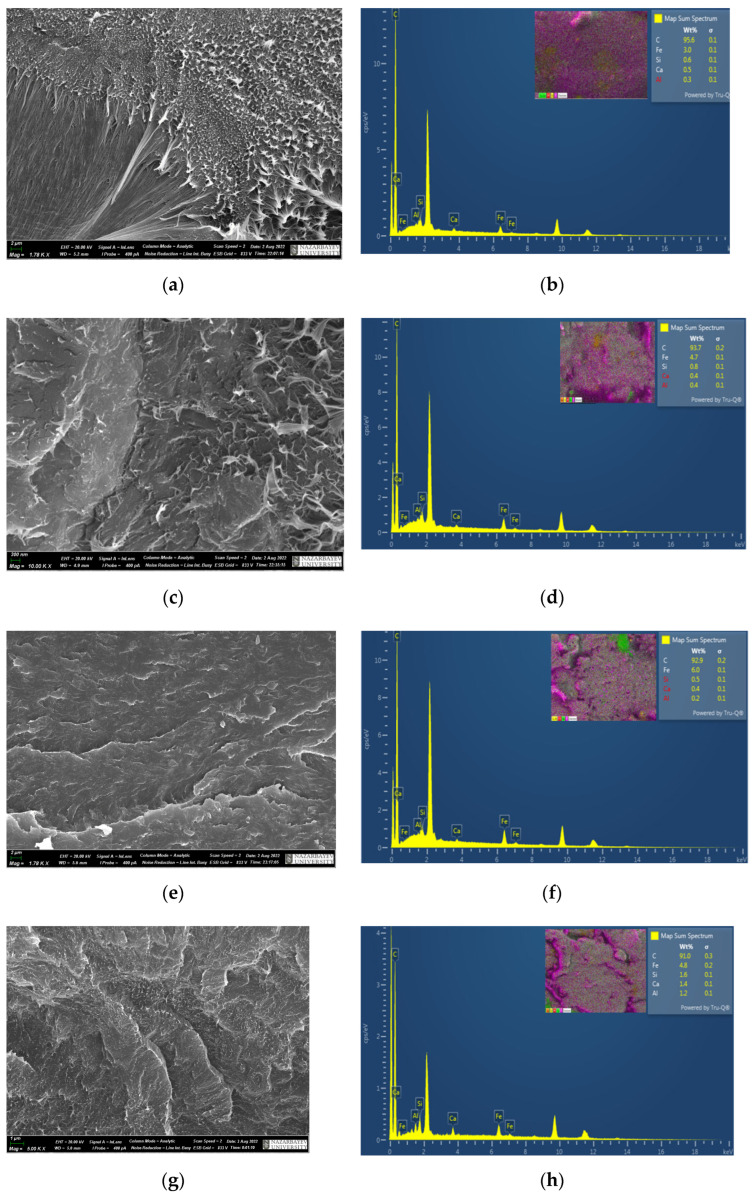
SEM images of the morphology of UHMWPE composite coatings with different diabase content: (**a**) structure of UHMWPE coating with 10 wt.%, (**b**) elemental analysis of the UHMWPE coating with 10 wt.%; (**c**) UHMWPE coating structure with 20 wt.%, (**d**) elemental analysis of the UHMWPE coating with 20 wt.%, (**e**) UHMWPE coating structure with 30 wt.%, (**f**) elemental analysis of the UHMWPE coating with 30 wt.%, (**g**) UHMWPE coating structure with 40 wt.%, (**h**) elemental analysis of UHMWPE coating with 40 wt.%.

Figure 4 shows SEM images of UHMWPE composite coatings with different contents (10, 20, 30 and 40 wt.%) of diabase and the corresponding element mapping spectra. SEM images combined with EDS element mapping were used to evaluate the dispersion analysis of fillers in the polymer matrix. As can be seen from the figure, for composite coatings with a diabase content of 10, 20, 30 and 40 wt.%, signs of agglomeration of the filler particles were not observed. Particles of diabase filler in the form of individual dots are completely embedded in the UHMWPE matrix (Figure 4) and uniformly distributed in UHMWPE, and this shows the excellent retention of composites in coatings. The diabase particles are not the center of the initiation of any cracks. As can be seen from the figures, the structure of composite coatings is uniform for all coating compositions.

### 3.3. Results of X-ray Phase Analysis of Powders and Coatings

**Figure 5:**
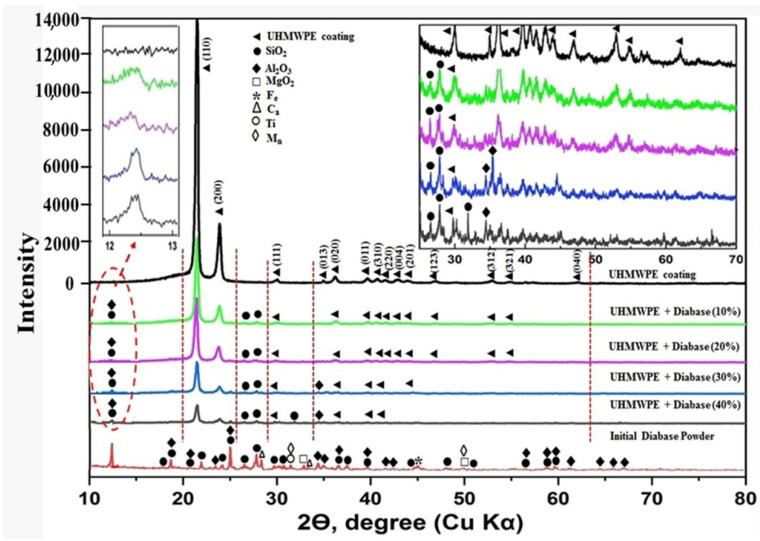
X-ray diffraction patterns of flame spraying of UHMWPE, UHMWPE + diabase within 10–40 wt.% and diabase powders.

Figure 5 shows X-ray diffraction patterns of flame spraying of UHMWPE, UHMWPE + diabase within 10–40 wt.% and diabase powder. Diffraction reflections typical of UHMWPE are visible. A similar diffraction pattern for UHMWPE was obtained in [25]. Peaks of diabase due to the complex chemical composition have a low intensity. It can be seen from the diffraction patterns that the gas-flame effect and the introduction of the mineral diabase filler do not adversely affect the UHMWPE polymer matrix. No shifts of diffraction lines are observed. UHMWPE has an orthorhombic lattice in accordance with [26]. It can be seen that the intensity of the diffraction line (110) of the phase of the initial coating from pure UHMWPE reaches 14,000; when diabase is added to the composition of 10–20%, the intensity of the diffraction line decreases to 5000; with an increase in the content of diabases to 40%, the intensity of the diffraction line of the polymer decreases to 1500. The intensity of UHMWPE peaks with an increase in the degree of filling, which is due to a decrease in the content of UHMWPE in the volume.

X-ray phase analysis showed that the degree of crystallinity practically does not change and remains constant within the error. The data on the degree of crystallinity of the coatings are given below in Table 2. It can be seen that the flame spraying of UHMWPE with the addition of diabase in the range of 10–40 wt.% does not significantly affect the degree of crystallization of the coating.

### 3.4. DSC Results—Analysis of Powders and Coatings

**Figure 6:**
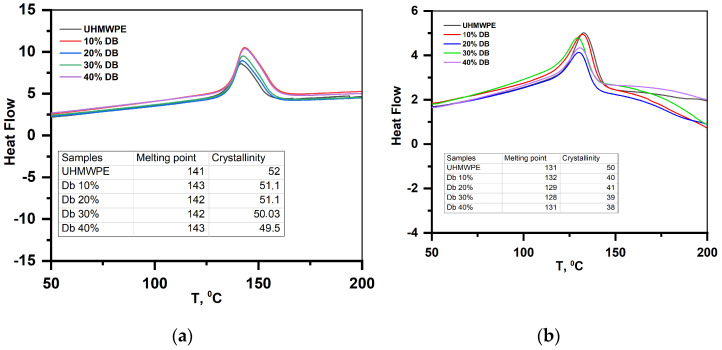
DSC analysis of the initial mixture of UHMWPE powders with diabase filler (**a**), composite coating of UHMWPE with diabase filler (**b**).

Figure 6 shows the results of DSC analysis of UHMWPE powders with diabase filler in the range of 10–40% of the total mass before coating (Figure 6a) and the results of DSC analysis of the coating obtained on the basis of these compositions (Figure 6b). According to the obtained results, the melting temperature of the mixture of UHMWPE powders containing 10–40% diabase was about 140 °C before coating and about 130 °C for the resulting coating. The degree of crystallinity of powder mixtures, according to DSC analysis, was about 50%. The degree of crystallinity of the coatings decreased by ~8%. The degree of crystallinity and melting point of UHMWPE depend on their initial thermal history, according to [14,27].

### 3.5. Results of IR Analysis of Coatings

**Figure 7:**
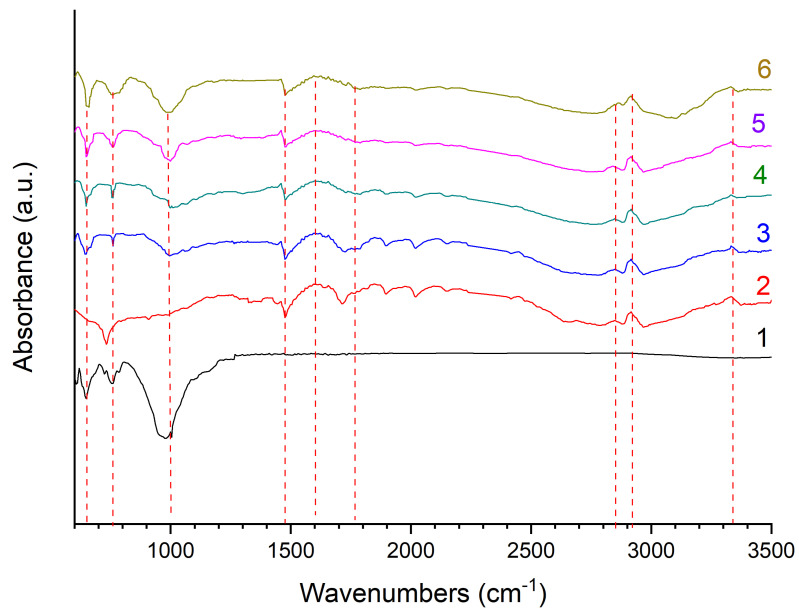
IR spectra: 1 diabase powder; 2 UHMWPE coating; 3, 4, 5, 6 UHMWPE coating with diabase filler 10, 20, 30, 40 wt.%.

In Figure 7, the IR spectra of diabase powders show that a small shoulder located at 550 cm^−1^ can be attributed to the bending vibration of Si-O-Al. The band observed at 646 cm^−1^ is due to the stretching vibration of Al-O [28,29]. In the Fourier transform IR spectra, peaks are observed at 743 and 780 cm^−1^, which can be attributed to symmetric Si-O-Si stretching vibrations and Si-Si stretching vibrations [30]. The broadband peak observed between 850 and 1250 cm^−1^ is associated with asymmetric stretching vibrations of Si-O-Si, Si-O-Al and Si-O bonds [28].

As shown, all coating samples have absorption peaks associated with C-H at 2932, 2856, 1476 and 728 cm^−1^, which correspond to methylene antisymmetric stretching vibrations, methylene symmetric stretching vibrations, methylene angle-changing vibrations and methylene vibrations, vibrational vibrations, respectively [31].

Small absorption peaks at 3340 cm^−1^ are probably associated with the hydroxyl group (-OH) or water absorbed in the samples. The band marked at ~1472 cm^−1^ is the absorption peak of C-C bonds, which characterize the structure of UHMWPE. The absorption peaks located in the range of 1750–1600 cm^−1^ may reflect the stretching vibrations of the C=O groups [32]. This is most likely due to the reaction of a small amount of UHMWPE with oxygen during sample preparation. It is noted that the FTIR spectra showed no significant changes or shifts in the main peaks for the original powders and coatings after deposition, indicating that the UHMWPE structure did not undergo obvious thermal degradation during the flame formation of the coating.

### 3.6. Results of Thermogravimetric Analysis of Powders and Coatings

**Figure 8:**
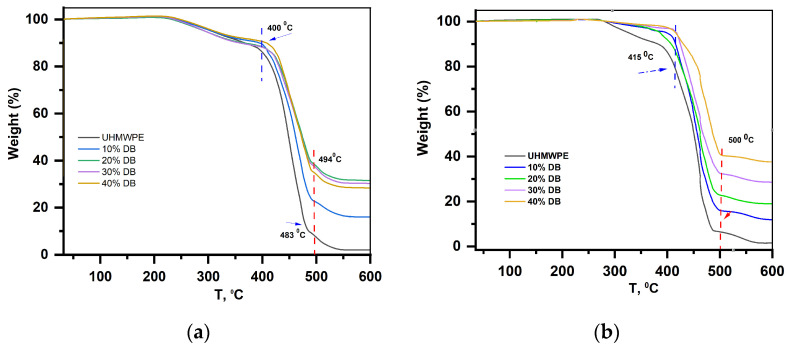
Thermograms of the initial mixture of UHMWPE and diabase powders (**a**), UHMWPE composite coating with diabase filler (**b**).

Figure 8a shows thermograms of the heat resistance of a mixture of UHMWPE with an admixture of diabase in the range of 10–40% of the total mass before coating obtained with these compositions. It can be seen that the UHMWPE mixture containing 10–40% diabase undergoes thermal decomposition in the temperature range of 415–500 °C. Figure 8b shows the thermograms of the UHMWPE composite coating with diabase filler in the range of 10–40%. From the comparison of the thermograms, it can be seen that the temperature at the beginning of the decomposition of the composite coating is 15 °C higher than that of the initial mixture. Previously, it was shown in [33,34] that the addition of filler keeps the thermal decomposition temperature.

### 3.7. Results of Studies of the Tribological Properties of Coatings

Table 2 shows the characteristics of UHMWPE coatings with different diabase content: degree of crystallinity (χ), coating surface roughness (Ra) and mass wear (∆m).

It can be seen from the table that with an increase in the degree of filling with diabase from 10 to 40%, the roughness increases from 1.237 to 4.311 µm and the wear resistance decreases with an increase in the diabase content. The introduction of diabase into UHMWPE leads to an increase in abrasive wear resistance in comparison to a coating of pure UHMWPE by several times.

**Figure 9:**
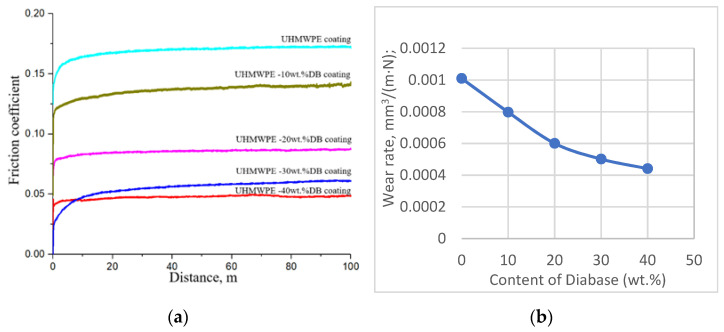
Variation in friction coefficient (**a**) and wear rate (**b**) dry sliding friction of UHMWPE and composites filled with diabase powders: UHMWPE, UHMWPE + diabase powders—10 wt.%, 20 wt.%, 30 wt.%, 40 wt.%.

Tests of coatings to determine the coefficient of friction showed that the addition of diabase leads to a noticeable decrease in the coefficient of friction (Figure 9a): from ~0.17 for a coating of pure UHMWPE to ~0.14 for a coating of UHMWPE 10 wt.% diabase, ~0.09 for UHMWPE 20 wt.% and ~0.06 for UHMWPE 30 wt.% and ~0.05 for UHMWPE coating 40 wt.% diabase.

Figure 9b shows the wear rate, from which it follows that the wear resistance of UHMWPE composites + n wt.% diabase increases when the matrix is filled. It can be seen that the UHMWPE + 40 weight composition is characterized by the highest wear resistance % diabase (2.2 times higher than pure UHMWPE). The minimum wear resistance is possessed by a sample, the proportion of filler that is minimal 10 wt.% (1.25 times higher compared to pure UHMWPE). With an increase in the content of diabase in UHMWPE, the wear rate decreases, which indicates an improvement in the wear-resistant characteristics of UHMWPE due to the addition of diabase.

**Figure 10:**
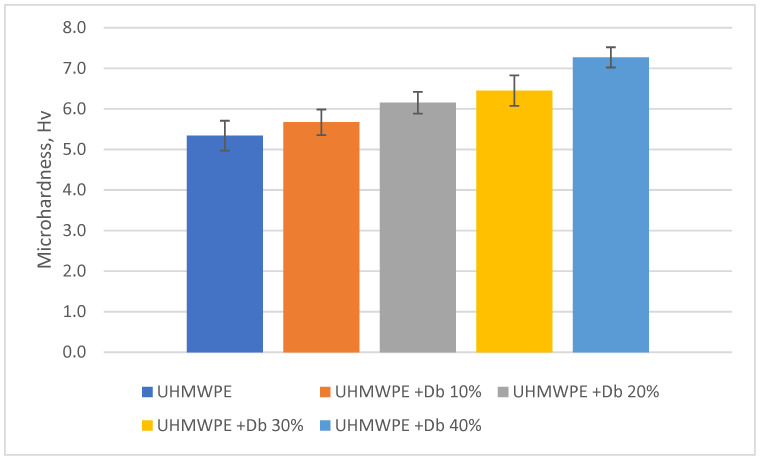
Changes in the microhardness of UHMWPE and composite coatings based on UHMWPE depending on the content of diabase filler.

Figure 10 shows the microhardness values of UHMWPE + diabase composite coatings with different filler content.

It can be seen that the microhardness of the coatings increases with an increase in the diabase content: 5.34 Hv for a coating of pure UHMWPE, 5.67 Hv for a coating of UHMWPE + diabase 10 wt.%, 6.15 Hv and 6.95 Hv for a coating of UHMWPE + diabase 20 and 30 wt.% and for coating UHMWPE + diabase 40 wt.%, the microhardness is 7.27 Hv. The increased values of the microhardness of the composite coating with an increase in the diabase content are apparently due to the relatively uniform distribution of diabase in the UHMWPE matrix, as well as the effective mixing of the composites and the envelopment of the filler particles by the polymer matrix, which, accordingly, contributes to good indentation.

## 4. Conclusions

Thus, this paper presents the results of studies of a composite coating of ultrahigh molecular weight polyethylene with a diabase filler obtained using flame spraying. Diabase 10 wt.%, 20 wt.%, 30 wt.% and 40 wt.% was chosen as a filler.

It is shown that the introduction of diabase into the composition of UHMWPE with a content of 10–40% of the total mass does not significantly affect the degree of crystallinity of the coating. According to XRD, the degree of crystallinity practically remains at the level of 60%, and according to DSC data, the degree of crystallinity of the coating does not significantly decrease with increasing diabase filler content: 50% for pure UHMWPE, 38% for UHMWPE +40% with diabase filler.

It has been established that the diabase filler does not have the same negative effect on the chemical structure of UHMWPE during flame spraying and coatings made of UHMWPE with filler do not undergo degradation during flame spraying.

The thermal stability of UHMWPE coating with diabase in the range of 10–40% was studied using thermogravimetric analysis. Based on the results obtained, it was found that the decomposition temperature of the coating is 15 °C higher than that of the original UHMWPE powder with a diabase content of 10–40%.

It has been established that with an increase in the volume of the filler, the wear resistance increases by more than 2 times compared to pure UHMWPE. The roughness increases from 1.237 to 4.311 µm with an increase in the degree of filling with diabase from 10 to 40%.

Studies have also shown that the addition of diabase leads to a noticeable decrease in the coefficient of friction of the coating: for a coating of pure UHMWPE~0.17; UHMWPE + diabase 10 wt.%~0.14; UHMWPE + diabase 20 wt.%~0.09; UHMWPE + diabase 30 wt.%, 0.06; and for coating UHMWPE + diabase 40 wt.%~0.05.

It is determined that the microhardness of the coatings increases with increasing diabase content: the microhardness for a coating of pure UHMWPE is 5.34 Hv; for a coating of UHMWPE + diabase 10 wt.% 5.67 Hv; for UHMWPE + diabase coating 20 and 30 wt.%, 6.15 Hv and 6.95 Hv; respectively, for the coating UHMWPE + diabase 40 wt.% is 7.27 Hv. 

## 5. Patents

Based on the results of the work carried out, we received a utility model, patent no. 7206, dated 3 March 2022: Powdered material for thermal spraying of polymer coatings. Ocheredko I.A., Skakov M.K., Tuyakbaev B.T., Erbolatuly D., Bayandinova M.B.

## Figures and Tables

**Table 1:** Chemical composition of diabase powder (wt.%).

Spectrum	O	Na	Mg	Al	Si	Ca	Ti	Fe	Total
Spectrum 1	41.40		8.03	2.08	24.06	10.82		13.61	100.00
Spectrum 2	46.87		8.91	0.98	24.24	8.67		10.33	100.00
Spectrum 3	39.10		8.46	1.14	26.79	11.69		12.82	100.00
Spectrum 4	51.71	0.77	8.12	4.85	18.49	4.52		11.53	100.00
Spectrum 5	47.29		8.39	0.93	23.72	8.96	0.58	10.12	100.00

**Table 2:** Mechanical properties and degree of crystallinity of coatings.

Coating Composition	χ, %	Ra, µm	Wear, ∆m, g		
			10 min.	20 min.	30 min.
UHMWPE	67	1.237	0.0131	0.0372	0.0542
UHMWPE 10% Diabase	60	3.656	0.0103	0.0258	0. 0351
UHMWPE 20% Diabase	60	3.805	0.0065	0.0201	0.0321
UHMWPE 30% Diabase	59	4.107	0.0045	0.0185	0.0303
UHMWPE 40% Diabase	60	4.311	0.0012	0.0132	0.0278

## Data Availability

Data will be made available on request.
